# Antibacterial Interactions of Ethanol-Dispersed Multiwalled Carbon Nanotubes with *Staphylococcus aureus* and *Pseudomonas aeruginosa*

**DOI:** 10.1021/acsomega.4c03044

**Published:** 2024-07-09

**Authors:** Mihaela Asaftei, Massimiliano Lucidi, Stefan Razvan Anton, Aikaterini-Flora Trompeta, Radu Hristu, Denis E. Tranca, Efstathios Fiorentis, Cristina Cirtoaje, Veronica Lazar, George A. Stanciu, Gabriella Cincotti, Paola Ayala, Costas A. Charitidis, Alina Holban, Paolo Visca, Stefan G. Stanciu

**Affiliations:** † Center for Microscopy-Microanalysis and Information Processing, National University of Science and Technology Politehnica Bucharest, 313 Splaiul Independentei, 060042 Bucharest, Romania; ‡ Department of Microbiology and Immunology, Faculty of Biology, Research Institute of the University of Bucharest, University of Bucharest, 060101 Bucharest, Romania; § Department of Science, Roma Tre University, Viale G. Marconi 446, 00146 Rome, Italy; ∥ NBFC, National Biodiversity Future Center, Piazza Marina 61, 90133 Palermo, Italy; ⊥ Research Lab of Advanced, Composite, Nano-Materials and Nanotechnology (R-NanoLab), School of Chemical Engineering, National Technical University of Athens, 9 Heroon Polytechniou, 15773 Athens, Greece; # Department of Engineering, Roma Tre University, Viale G. Marconi 446, 00146 Rome, Italy; ¶ Faculty of Physics, University of Vienna, Boltzmanngasse 5, A-1090 Vienna, Austria

## Abstract

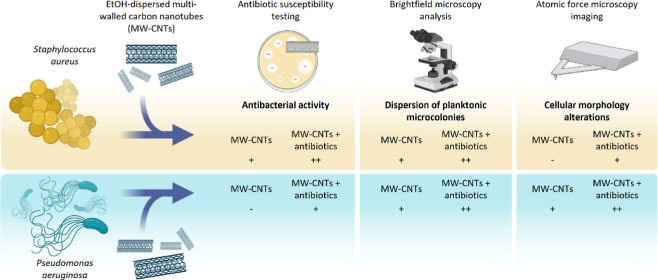

Infectious diseases are acknowledged as one of the leading causes of death worldwide. Statistics show that the annual death toll caused by bacterial infections has reached 14 million, most of which are caused by drug-resistant strains. Bacterial antibiotic resistance is currently regarded as a compelling problem with dire consequences, which motivates the urgent identification of alternative ways of fighting bacteria. Various types of nanomaterials have been reported to date as efficient antibacterial solutions. Among these, carbon-based nanomaterials, such as carbon nanodots, carbon graphene oxide, and carbon nanotubes (CNTs), have been shown to be effective in killing a wide panel of pathogenic bacteria. With this study, we aim to provide additional insights into this topic of research by investigating the antibacterial activity of a specific type of multiwalled CNTs, with diameters from 50 to 150 nm, against two representative opportunistic pathogens, i.e., the Gram-positive bacterium *Staphylococcus aureus* and the Gram-negative bacterium *Pseudomonas aeruginosa*, both included among the top antibiotic-resistant pathogens. We also test the synergistic effect of CNTs with different antibiotics commonly used in the treatment of infections caused by *S. aureus* and/or *P. aeruginosa*. Additionally, a novel approach for quantitatively analyzing bacterial aggregation in brightfield microscopy images was implemented. This method was utilized to assess the effectiveness of CNTs, either alone or in combination with antibiotics, in dispersing bacterial aggregates. Finally, atomic force microscopy coupled with a newly devised image analysis pipeline was used to examine any potential morphological changes in bacterial cells following exposure to CNTs and antibiotics.

## Introduction

Antibiotics are effective antibacterial drugs, but their wide use is invariably associated with the emergence of bacterial resistance, which is now recognized as a serious problem in both clinical and community settings.^1,2^ The emergence of drug-resistant bacterial strains has raised the need to exploit alternative strategies to counteract infectious diseases. Nanotechnology offers new promising approaches to synthesizing novel compounds with antibacterial and antibiofilm properties, and a wide palette of nanomaterials has been studied to date to thoroughly understand their antimicrobial properties.^3−5^ Besides their stand-alone use, solutions building on the combined effects of nanomaterials and antibiotics have also been the focus of interest in many studies.^6−8^ Antibiotic nanoparticle formulations can offer several advantages over conventional administration and delivery methods, including the ability for drug delivery to a specific site such as an intracellular infection. Nanomaterials can also be exploited to facilitate the sustained release of an antibiotic, minimizing dosing regimens.^9,10^ Furthermore, nanomaterials can mask the entrapped drug,^11^ reducing systemic toxicity induced by conventional administration of the free drug.

Among the various nanomaterials endowed with antimicrobial properties, carbon-based nanomaterials, including carbon nanodots, graphene oxide, carbon onions, carbon nanotubes (CNTs), and others have been demonstrated as being highly effective in killing pathogenic bacteria, based on diverse mechanisms of action.^8,12,13^ CNTs are graphite sheets whose layers appear as a rolled up, nonstop unbreakable hexagonal-like mesh structure with the carbon molecules positioned at the apexes of the hexagonal structures.^14^ CNTs can be synthesized in different sizes, and depending on the dimensions, they account for diverse mechanisms of antibacterial activity. For example, short CNTs are known to pierce bacterial cell walls and membranes, while long CNTs are known to wrap around the bacteria, interfering with their vital functions. Existing knowledge about the interactions between CNTs and various bacterial cells has been reviewed in the past.^8,12,13,15^

Despite consistent efforts of the scientific community to achieve a complete picture of the antibacterial activity of CNTs, there are vast aspects still poorly understood. Part of the reasons behind this relates to the wide variety of CNTs’ configurations, in terms of morphology, functionalization, and various physicochemical properties that highly differ among distinct CNT variants. With this study, we aim to contribute to this gap by investigating the antibacterial activity of ethanol (EtOH)-dispersed multiwalled CNTs of diameters ranging from 50 to 150 nm, against the opportunistic pathogens *Staphylococcus aureus* and *Pseudomonas aeruginosa*, characterized by extremely diverse surface structures likely responsible for different physical interactions with CNTs. The reason for investigating EtOH-dispersed CNTs refers to the typical solvents used for these nanomaterials. While water is the desired choice for assessing the antibacterial action of CNTs, the hydrophobic nature of these nanomaterials causes them to repel water, resulting in clustering. This clustering poses a challenge when assessing the antibacterial activity of the nanosized CNTs at the individual level. Solutions for dispersing CNTs in water do exist,^16−18^ for example, by using surfactants, functionalization, or polymer wrapping, but these interfere with the intrinsic antibacterial activity of the CNTs, which can hardly be evaluated upon such treatments. The solvents that are commonly used for dispersing CNTs^16,19^ include: *N*-methyl-2-pyrrolidone (NMP), which is effective in breaking down the agglomerates and promoting the dispersion of individual nanotubes; dimethylformamide (DMF), known to achieve good dispersion by breaking down the van der Waals forces between nanotubes; dimethyl sulfoxide (DMSO), known for its ability to solubilize a wide range of materials; but also isopropanol and EtOH, two alcohols that are polar solvents and can help break down CNT agglomerates. Our interest lies in evaluating EtOH-dispersed CNTs, given potential hybrid applications in wound dressings. These play a crucial role in the management of wounds,^20,21^ with key roles in infection prevention, moisture control, promotion of healing, pain management, minimization of scarring, absorption of exudate, protection from mechanical trauma, or comfort and aesthetics. While wound dressings of many types have been reported to date, the scientific community is still in search of more effective variants.^21^ Alcohols have been used as dressing for wounds from the oldest times.^22^ However, EtOH, which is a common antiseptic and disinfectant used to clean and disinfect wounds, is not routinely used in wound dressings, given that prolonged EtOH exposure can impair the proliferative response during healing, and cause delays in epithelial coverage, collagen synthesis, and blood vessel regrowth.^23^ However, recent evidence on some of the advantages of EtOH-treated or -embedding wound dressings and scaffolds^24−28^ has started to emerge. Since wound dressings with CNTs^29−31^ and with antibiotics^32−34^ have been widely discussed to date, both presenting important advantages for specific applications, we find it useful to investigate potential synergies between these three antibacterial agents: CNTs, EtOH, and antibiotics. As far as we know, there is currently limited knowledge on this topic.

This work focuses on two bacterial species (i.e., *S. aureus* and *P. aeruginosa*) used as experimental models of pathogens. *S. aureus* is a Gram-positive bacterium whose envelope is composed of a thick peptidoglycan layer surrounding the cytoplasmic membrane. It is responsible for a wide variety of clinical manifestations in animals and humans, including both community and hospital-acquired infections. Treatment remains challenging to manage due to the emergence of multidrug-resistant strains such as MRSA (methicillin-resistant *S. aureus*).^35,36^ MRSA caused more than 100,000 deaths attributable to antimicrobial resistance in 2019 only.^37^*P. aeruginosa* is another opportunistic pathogen endowed with natural and acquired antibiotic resistance, responsible for causing important illnesses in humans.^38,39^ As a Gram-negative species, *P. aeruginosa* is enveloped by two membranes separated by the periplasmic space. Despite their structural differences, both *S. aureus* and *P. aeruginosa* are classified as ESKAPE pathogens (Enterococcus faecium, *S. aureus*, *Klebsiella pneumoniae*, *Acinetobacter baumannii*, *P. aeruginosa*, and *Enterobacter species*) because of their multidrug resistance, which allows them to elude the activity of last-resort antibiotics.^40,41^

In this work, we attempted to expand the current knowledge on the antibacterial effects of CNTs, either as stand-alone weapons or as therapeutic tools synergizing with antibiotics. In brief, we assessed the antibacterial activity of CNTs against *S. aureus* and *P. aeruginosa* by disk diffusion (Kirby Bauer) and microdilution methods, followed by viable counts. We also assessed the synergistic effect of CNTs and different antibiotics routinely utilized for disk diffusion testing of *S. aureus* and *P. aeruginosa*. Furthermore, we implemented a pipeline to assess from brightfield microscopy images how CNTs and two antibiotics, ciprofloxacin (CIP) and gentamicin (CN), impact the dispersion of bacterial aggregates in suspension. Last, we employed atomic force microscopy (AFM), augmented by statistical analysis, to screen for potential morphological changes in the bacterial cells upon their interaction with the CNTs and the two antibiotics at focus. The presented results add knowledge to the previous efforts devoted to painting a complete picture of the antibacterial effects of CNTs.

## Materials and Methods

### Bacterial Strains and Culture Media

*P. aeruginosa* ATCC 27853 and *S. aureus* ATCC 25923 were obtained from the American Type Cell Collection (ATCC) and routinely grown in BBL Mueller Hinton II (cation-adjusted) broth (MHB, Becton Dickinson) or MHB supplemented with 1.5% (w/v) agar (MHA) at 37 °C. Antibiotic discs were purchased from BioMerieux (Marcy l’Étoile, France). CIP and CN powders were purchased from Merck KGaA (Darmstadt, Germany).

### CNT Synthesis

The CNTs of the current study have been synthesized in the Research Lab of Advanced, Composites, Nanomaterials and Nanotechnology (R-NanoLab) of the National Technical University of Athens, through the chemical vapor deposition (CVD) method, in a custom-made vertical CVD reactor that enables the continuous production of the CNTs. The reactor consists of a vertical tubular furnace that reaches up to 750 °C and is equipped with specific glassware and mechanical components for the inlet (supply of inert gas and liquid precursor) and outlet of the system (product collection and emissions exhaust). The furnace, as the main body of the reactor, offers the necessary thermal energy for the catalytic decomposition of the carbon precursor and the formation of the CNTs on the catalytic substrate.^42,43^ Within the furnace, a metallic tube from stainless steel is placed, in which the reaction takes place. A hybrid floating/supported catalyst approach has been utilized for the growth of the CNTs of this work. A reaction mixture of 20% wt. ferrocene (C_10_H_10_Fe) in EtOH (C_2_H_6_O), prepared by ultrasonication for 20 min, served as the liquid carbon precursor. This was introduced into the active zone (*T* = 700 °C) of the reactor dropwise through a separating cylindrical funnel, with the aid of gravity and nitrogen (N_2_) flow of 300 mL/min. The N_2_ ensured inert conditions in the system and carried the reaction mixture droplets. During the reaction, EtOH was decomposed, and the carbon atoms and radicals started forming carbon nanostructures on the reactor walls, made from stainless steel (including Fe, Cr, Mo, and Ni, which favor the growth of CNTs). The outlet was redirected within a fume hood, after getting through a scrubber, to collect the organic byproducts, soot, and other emissions. After the reaction was completed, the system was left to cool down at room temperature and then the resulting CNT product was collected from the bottom of the reactor, in a powder form, that was ground down to fine particles.

### CNT Characterization by Transmission Electron Microscopy

A FEI CM20 high-resolution transmission electron microscope with a thermionic gun LaB6 operating at 200 kV was used to determine the size and morphology of the CNTs, with magnification starting from ×64,000 (point resolution: 0.27 nm; information limit: 0.18 nm) at brightfield. To examine the CNT samples, dispersions in pure EtOH were prepared with successive dilutions, and one drop of the most dilute dispersion was placed on a carbon-coated Cu grid that allowed the solvent to evaporate.

### Evaluation of CNT Dispersion Stability

A specific amount of CNTs (0.1 mg) was dispersed through ultrasonication in 10 mL of EtOH in test tubes immersed in an ultrasonic bath for 1 h; a similar procedure has been implemented using distilled water, for comparison purposes.

### CNT Preparation for Antibiotic Susceptibility Testing

The powder of CNTs was dispersed in pure EtOH at a final concentration of 2 mg/mL. The resulting dispersion was stored at room temperature and vigorously vortexed for 5 min before use to ensure the complete dispersion of any CNT particles.

### Qualitative CNT Susceptibility Assays

The antibacterial activity of the CNTs was assessed by using the Kirby–Bauer disk diffusion assay. Briefly, 18 h cultures in MHB were washed and diluted in saline to an optical density at 600 nm (OD_600_) = 0.1 (corresponding to ca. 2 × 10^8^ colony-forming units (cfu)/mL and 4 × 10^7^ cfu/mL for *P. aeruginosa* ATCC 27853 and *S. aureus* ATCC 25923, respectively) and then seeded with a sterile swab on the surface of MHA. Sterile 6 mm blank disks (Thermo Fisher-Oxoid) soaked with 20 μL of a 2 mg/mL dispersion of CNTs was deposited on the agar surface, and the growth inhibition halo (hereafter, zone of inhibition, ZOI) was detected after 18 h of incubation at 37 °C. Blank disks soaked with 20 μL of EtOH were used as control.

### Bacterial Growth Curves in the Presence of CNTs

18 h bacterial cultures in MHB were diluted to ca. 5 × 10^5^ cfu/mL in a 96-well microtiter plate containing a final volume of 200 μL of MHB supplemented with 2-fold serially diluted concentrations of CNTs (ranging from 0.25 to 0 mg/mL) or the same volume of EtOH [ranging from 12.5 to 0% (v/v)], used as control. Bacterial cells were incubated for 60 h at 37 °C and the OD_600_ of the cultures was measured every 2 h by using the Spark 10 M (Tecan) microplate reader. OD_600_ of bacterial-free media was used as blank and subtracted to the samples to eliminate the OD_600_ contribution of CNTs.

### Evaluation of CNTs’ Effects on Bacterial Viability

The antibacterial activity of CNTs was assessed by the macrodilution method (Clinical Laboratory Standards Institute, 2021) with few modifications. Briefly, *S. aureus* ATCC 25923 was grown for 18 h in MHB and then diluted to obtain ca. 5 × 10^5^ cfu/mL in 3 mL of MHB supplemented with 2-fold serially diluted concentrations of CNTs (ranging from 0.25 to 0 mg/mL) or the same volume of EtOH [ranging from 12.5 to 0% (v/v)], used as control, and incubated at 37 °C under vigorous shaking (i.e., 220 rpm). After 16 h, cells were 10-fold diluted in saline and 5 μL of this bacterial suspension was deposited on MHA. CFUs were determined after 24 h incubation at 37 °C.

The broth microdilution method was used to determine the minimum inhibitory concentration (MIC; Clinical and Laboratory Standards Institute, 2021). Briefly, bacteria were grown for 18 h in MHB and then diluted to obtain ca. 5 × 10^5^ cfu/mL in 200 μL of MHB supplemented with 2-fold serially diluted concentrations of CNTs (ranging from 0.25 to 0 mg/mL) or the same volume of EtOH [ranging from 12.5 to 0% (v/v)], using 96-well microtiter plates. Plates were incubated for 24 h at 37°. The MIC of CNTs was defined as the lowest concentration that completely inhibited bacterial growth as detected by the unaided eye (Clinical Laboratory Standards Institute, 2021), and OD_600_ measurements of 24 h cultures in MHB were performed for confirmation.

### Interactions between CNTs and Antibiotics

The interactions between CNTs and a panel of antibiotics have been tested by disk-diffusion assay. Briefly, 18 h cultures of *P. aeruginosa* ATCC 27853 and *S. aureus* ATCC 25923 in MHB were diluted to OD_600_ = 0.1 and seeded on the surface of MHA. Antibiotic-containing disks were then positioned on the plate surface. A 20 μL solution of EtOH-dispersed CNTs (at a concentration corresponding to 0.5 × MIC) or pure EtOH has been added to antibiotic-containing disks. After 24 h of incubation at 37 °C, the ZOI for antibiotics alone (ZOI_ant_) and antibiotics with the addition of EtOH (ZOI_ant+EtOH_) or EtOH-dispersed CNTs (ZOI_ant+CNTs_) were determined. The percentages of growth inhibition were calculated according to the following equations
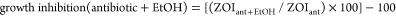
1
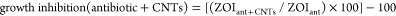
2

### Microscopy Sample Preparation

CIP and CN antibiotics were 2-fold serially diluted in CNT-supplemented MHB (0.5 mg/mL) to achieve concentrations ranging from 2 to 0.0009 μg/mL. Then, overnight-grown bacterial cultures were applied to all wells except negative control to obtain a final bacterial concentration of 5 × 10^5^ cfu/mL in each well. After inoculation, the plates were incubated for 24 h at 37 °C. The broth microdilution method was performed in duplicate for each bacterial isolate. A drop of the bacterial suspension from wells where growth was visible by the unaided eye was taken from the 96-well microtiter plates at the end of the broth microdilution assay and dispersed on the surface of a glass cover slide. After air-drying at room temperature, samples were fixed at the flame, stained with Giemsa for 15 min, washed, and visualized by brightfield microscopy. The samples for AFM studies were prepared similarly, except for the staining step.

### Brightfield Microscopy

For brightfield microscopy imaging, a Leica DM 3000 microscope, equipped with an MC 190 HD camera based on an s-CMOS sensor with reduced noise factor, was used. Images were collected using a Leica N Plan L 50×/0.5 objective, and the field diaphragm was tuned to avoid vignetting effects. The images were acquired at a digital resolution of 3648 × 2736 pixels, with a pixel size of 60 nm.

### Atomic Force Microscopy

An AFM system (NX10, Park Systems, Suwon, Korea) operated in non-contact mode was employed to investigate the surface topography of the synthesized samples. An aluminum-coated probe (PPP-NCHR 10 M, Nanosensors, Switzerland), with a radius of curvature <7 nm, and a tip length and width of 125 and 30 μm, respectively, was used. The resonant frequency of this probe was 330 kHz. The calibration of the equipment was carried out using an HS 100-MG probe (BudgetSensors, Innovative Solutions Bulgaria, Ltd.) as a standard sample composed of silicon dioxide structure arrays on a 5 × 5 mm silicon chip. Following data acquisition, the images were corrected for stage drift and other common AFM artifacts with the open-source Gwyddion software.^44^

### Statistical Analysis

Statistical analysis was performed using the GraphPad Prism software (GraphPad Software, Inc., La Jolla, CA). Differences having a *p*-value of ≤0.05 were considered statistically significant. All microbiological experiments were repeated three times. All replicates shown are biological replicates.

## Results and Discussion

### Morphological Characterization of CNTs

**Figure 1:**
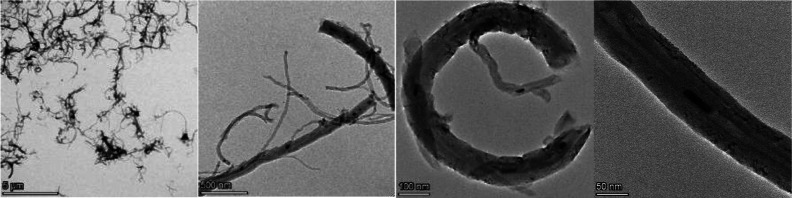
TEM images of synthesized CNTs.

TEM was used to assess the morphology of synthesized CNTs, revealing nanostructures with diameters in the range of 50 to 150 nm, thick walls, and a hollow core (Figure 1). Due to the synthetic method that was carried out (addition of catalyst in the reaction mixture and growth of CNTs on the catalytic substrate), the excess of catalyst (mainly Fe particles) is evident in the current nanostructures, as depicted in the TEM images, where the catalytic particles are shown as black elongated dots embedded in the CNTs’ internal structure. The sample tested was inert, without any surface functionalization or addition of functional groups.

### Dispersion Stability of CNTs in EtOH

**Figure 2:**
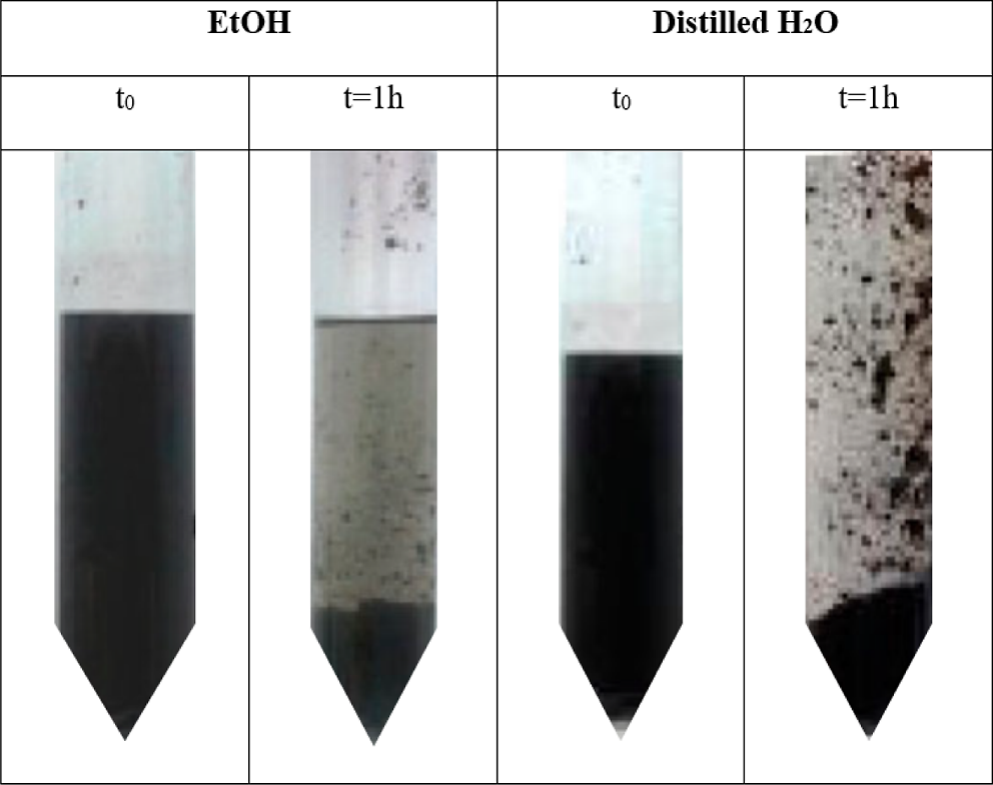
Dispersion test of CNTs in EtOH and distilled H_2_O.

To determine the dispersion stability of the synthesized CNTs in EtOH and distilled water, a dispersion test was performed.^45^ Distilled water is considered a neutral solvent since, among all the available solvents, it has the least impact on bacteria. However, as evident from the dispersion test results, pristine CNTs in distilled water did not exhibit a stable dispersion after 1 h of sonication, instead settling along with agglomerates on the walls of the tube. On the other hand, dispersion in EtOH was found to be more effective for the dispersion of CNTs 1 h after ultrasonication (Figure 2). In this condition, although sediment could be seen, the supernatant consisted of a homogeneous dispersion of CNTs, with few agglomerates. This test proves that EtOH is a more appropriate solvent for antibacterial testing compared to distilled water since a stable and homogeneous dispersion can be achieved during the experimental procedure.

### Antimicrobial Activity of CNTs

Kirby–Bauer assays showed that the CNTs evaluated at a concentration of 2 mg/mL proved a low antimicrobial effect against *S. aureus* and *P. aeruginosa*. The obtained ZOI diameters of the CNTs were comparable to the EtOH control (approximately 22 mm) (Figure S1). The limited inhibitory effects of the CNTs may be attributed to their partial entrapment within the disk fiber, as demonstrated by the restricted diffusion of CNTs in the agar plates (Figure S1).

**Figure 3:**
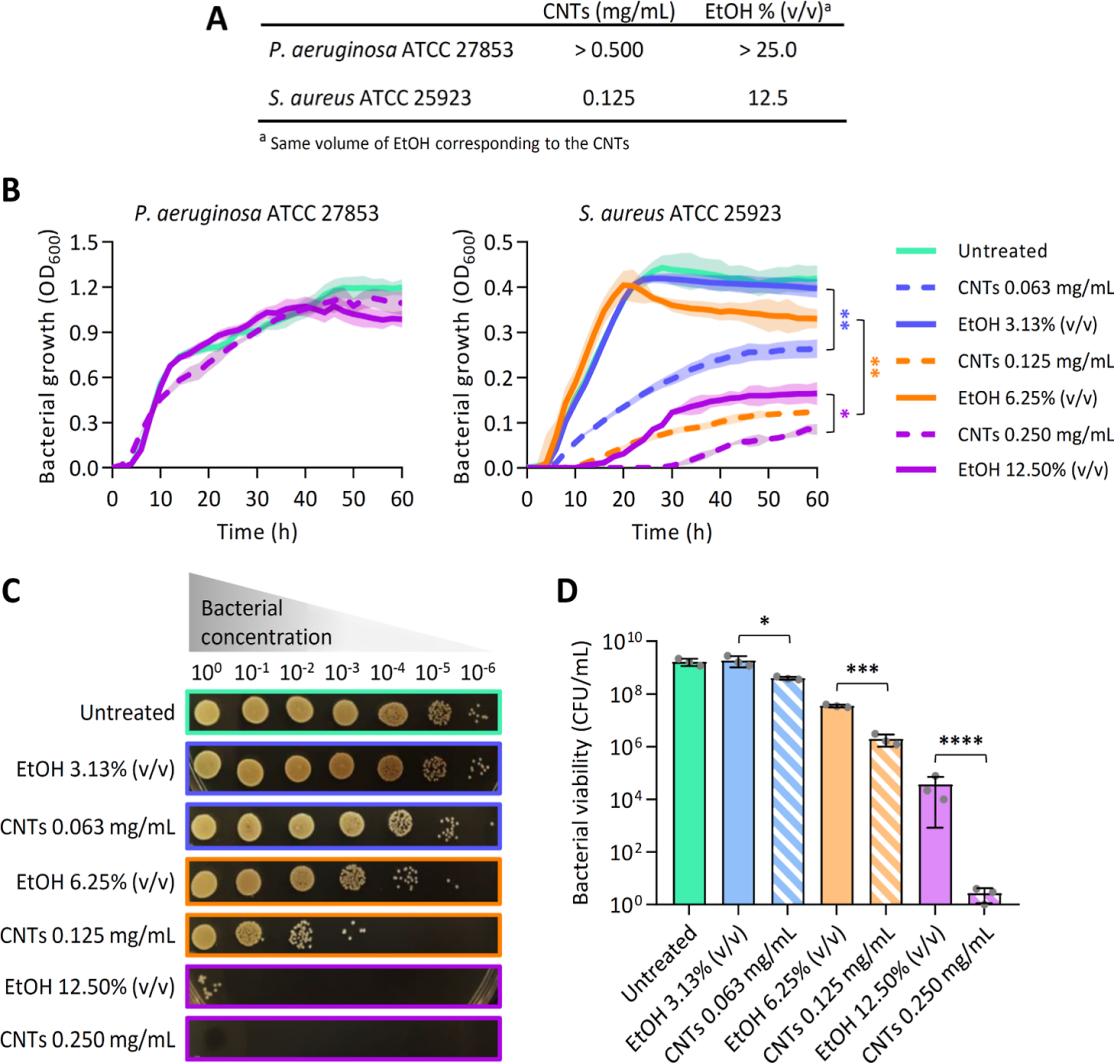
CNTs activity against *P. aeruginosa* and *S. aureus*. (A) MIC of CNTs. Due to the high volumes of CNTs tested (dissolved in EtOH), an equivalent volume of EtOH was used as a control. For instance, the maximum concentration of tested CNTs (0.5 mg/mL) corresponds to the addition of EtOH at a final concentration of 25% (v/v). (B) Growth curves of *P. aeruginosa* and *S. aureus* in MHB supplemented with the indicated concentrations of CNTs. MHB supplemented with the equivalent volume of EtOH was employed as a control. Asterisks represent statistically significant differences between the bacterial growth at 60 h in MHB supplemented with the indicated concentration of CNTs and the equivalent volume of EtOH (*p < 0.05; **p < 0.01). Data are the mean of three independent experiments ±standard deviation (SD), indicated as shaded areas. (C,D) *S. aureus* viability after 16 h of treatment with the indicated concentration of CNTs and EtOH. Asterisks in (D) represent statistically significant differences between the bacterial viability at the indicated concentration of CNTs and the equivalent volume of EtOH used to assay the CNTs (*p < 0.05; ***p < 0.001; ****p < 0.0001). Data in (D) are the mean of three independent experiments ±SD. *p* values in (B,D) were calculated using the unpaired *t*-test.

However, MIC determination revealed a difference in the antimicrobial potential of the evaluated CNTs, depending on the tested strain. The MIC of the CNTs against *P. aeruginosa* was higher than 0.5 mg/mL, comparable to the EtOH control. Conversely, we observed a lower MIC (0.125 mg/mL) for *S. aureus* compared to the EtOH control [MIC = 12.5% (v/v)], indicating a 2-fold difference (Figure 3A).

The analysis of the growth curve and CFU counts supports these results, showing a significant decrease in *S. aureus* viability in the presence of CNTs utilized at a concentration of 0.125 mg/mL, while no growth was obtained for this strain when a concentration of 0.250 mg/mL was used (Figure 3B–D).

A discussion of the above results can be summarized as follows: for a preliminary assessment of the antibacterial effect of CNTs, a disk-diffusion assay was performed on *S. aureus* and *P. aeruginosa* (Figure S1). For both bacterial species, a similar ZOI generated by EtOH-dispersed CNTs and EtOH alone was observed, suggesting that CNTs may not have a significant antibacterial effect by itself (Figure S1). However, agar diffusion tests, such as *E*-test and disk-diffusion assays, typically suffer from relative instability in maintaining concentration gradients of high-molecular-weight compounds (e.g., colistin, polymyxin B) and water-insoluble substances (e.g., polyphenolic compounds) in agar plates.^46−50^ Additionally, there is a risk of compounds becoming trapped in the disk fiber, which presents a limitation against the utilization of such systems.^46−50^ Therefore, isolates that were considered resistant to the action of these antimicrobials resulted in being susceptible when tested with reference tests, such as broth dilution methods.^46−50^ Since the tested CNTs are high-molecular-weight compounds that are water-insoluble and many particles remain trapped in the disk, we hypothesized that a low diffusion rate in the agar plates occurred.

To obtain more reliable results than disk diffusion assay, CNTs’ activities against *S. aureus* and *P. aeruginosa* was evaluated by the microdilution method. This approach confirmed that CNTs are ineffective against *P. aeruginosa* (MIC > 0.5 mg/mL) but revealed a slight antistaphylococcal activity, with an MIC of 0.125 mg/mL, compared to the MIC of EtOH alone (Figure 3A). Although the difference of a single MIC dilution between the compounds and the solvent alone (i.e., MIC of CNTs vs EtOH alone) should not be taken into consideration as predictive of a real antibacterial activity according to the CLSI guidelines, the difference between the MIC of CNTs and EtOH pushed us to investigate if it was correlated with a real interference with *S. aureus* growth. To further investigate this phenomenon, the bacterial growth of *P. aeruginosa* and *S. aureus* challenged with different concentrations of CNTs was monitored over time. Coherently with the results obtained with the microdilution method, no interference in the growth of *P. aeruginosa* cells was observed (Figure 3B). Moreover, the growth rate of *S. aureus* in the presence of CNTs presented a slight, despite significant, decrease compared to EtOH alone, used as control (Figure 3B). To consolidate the antistaphylococcal activity of CNTs, viable counts of *S. aureus* cells treated with CNTs were performed. The number of detected CFU of the bacterial cultures treated with CNTs was found to be significantly lower than those treated with EtOH alone (Figure 3C,D), corroborating the previous data and suggesting that CNTs had a bactericidal activity at the highest concentrations assayed (0.250 mg/mL), decreasing the number of viable *S. aureus* initially inoculated (ca. 10^5^ cfu/mL). Nevertheless, while the toxicity of these CNTs for human cells has not been tested in this study, other types of CNTs have been found to exhibit toxicity at the concentrations assayed (within the range of mg/mL) for various human cell lines,^51,52^ possibly narrowing the use of these CNTs in the clinical practice as antistaphylococcal compounds.

### Synergistic Activity of CNTs with Antibiotics

**Figure 4:**
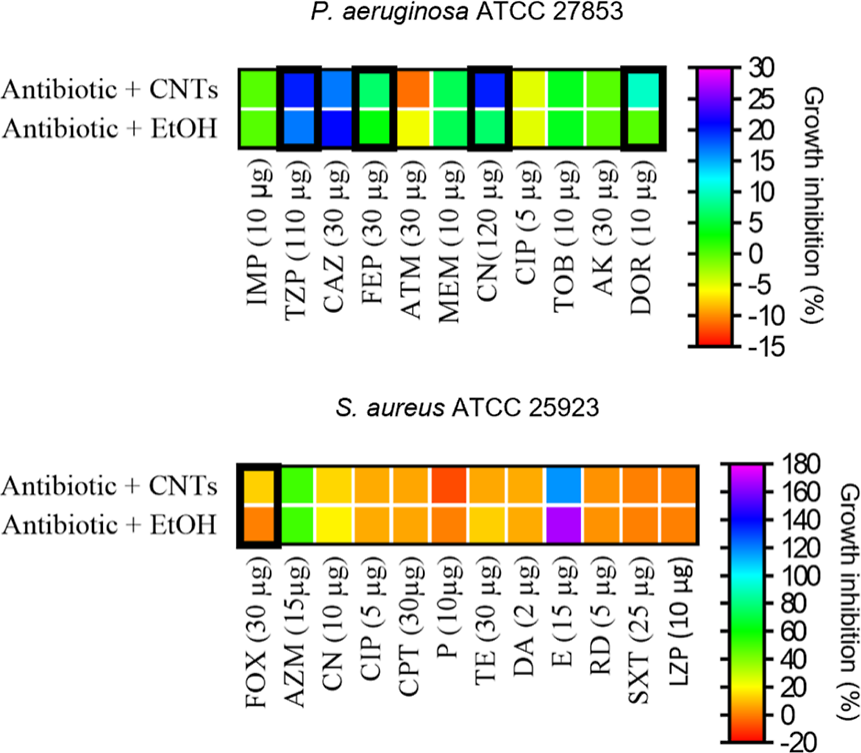
Disk-diffusion testing for the combination of antibiotics and EtOH-dispersed CNTs or EtOH. The percentage of growth inhibition was calculated according to eqs 1 and 2. Negative values indicate growth promotion compared to the control of the antibiotic alone. Black rectangles indicate conditions where ZOI_ant+CNTs_ > ZOI_ant+EtOH_, suggesting solvent-independent synergistic activities between CNTs and the tested antibiotics. Abbreviations: imipenem (IMP); piperacillin/tazobactam (TZP); ceftazidime (CAZ); cefepim (FEP); aztreonam (ATM); Meropenem (MEM); gentamicin (CN); ciprofloxacin (CIP); tobramycin (TOB); amikacin (AK); doripenem (DOR); cefoxitin (FOX); azithromycin (AZM); ceftarolin (CPT); penicillin G (P); tetracycline (TE); clindamycin (DA); erythromycin (E); rifampicin (RD); trimethoprim/sulfamethoxazole (SXT); levofloxacin (LZD). The antibiotic dosage for each disk is reported in brackets.

The use of CNTs in combination with antibiotics has important potential advantages for combating antibiotic resistance. For example, by lowering the required antibiotic dosage, while maintaining or increasing the antibiotic effectiveness, CNTs can reduce the selective pressure on bacteria to develop resistance. The interaction between CNTs and antibiotics in the fight against bacteria is, however, a complex and multifaceted topic, and the current body of understanding is still limited. The outcomes of combining CNTs with antibiotics can vary depending on several factors, including the type of CNTs, the type of antibiotics, and the specific bacteria involved.^8^ Motivated by this, we investigated the synergistic interaction of the CNTs with a panel of antibiotics routinely utilized for disk-diffusion testing of *S. aureus* and *P. aeruginosa*. For this purpose, a solution of EtOH-dispersed CNTs (or EtOH, used as control) has been directly added to antibiotic-containing disks. The resulting ZOIs were compared to those obtained with antibiotics alone (Tables S1 and S2 and Figure 4).

In *P. aeruginosa*, the antibiotics piperacillin/tazobactam (TZP), cefepim (FEP), gentamicin (CN), and doripenem (DOR) exhibited a synergistic effect with CNTs at subinhibitory concentrations (0.5 × MIC; Table S1 and Figure 4). In *S. aureus*, we have observed a synergistic interaction of the CNTs at 0.5 × MIC with cefoxitin (FOX; Table S2 and Figure 4). Interestingly, while the disk diffusion assay used to assess CNTs’ activity alone did not reveal any antibacterial effect (Figure S1), combining them with antibiotics highlighted some synergistic activities, suggesting a moderate CNTs’ diffusion on the plate making visible CNTs’ antibacterial effects. These preliminary results indicate a potential avenue for additional research through a checkerboard assay to examine the extent of synergistic activity between CNTs and antibiotics that have displayed enhanced efficacy.^53^

### Effects of CNTs on Bacterial Planktonic Aggregates

During planktonic growth in liquid cultures, bacteria can assemble into densely packed cell aggregates. This form of bacterial aggregation was found to play a role in resistance to antibiotics,^54^ microbe–host cell interaction,^55^ and expression of virulence factors.^56^ Although numerous studies have explored the antibacterial properties of various antibiofilm agents, there is a significant gap in understanding how antimicrobials affect cell aggregation during planktonic growth. To evaluate the efficacy of CNTs as dispersal agents, we treated *S. aureus* and *P. aeruginosa* cells with a subinhibitory concentration of CNTs (at 0.5 × MIC), either individually or in combination with antibiotics, and subsequently stained their planktonic forms with Giemsa dye.

From the 12 antibiotics tested in the previous section, we specifically selected CIP and CN, each with distinct mechanisms of action. CN is an antibiotic belonging to the aminoglycoside class that inhibits bacterial protein synthesis, leading to the death of the bacteria.^57^ On the other hand, CIP is an antibiotic belonging to the fluoroquinolone class. It works by inhibiting bacterial DNA gyrase and topoisomerase IV, enzymes involved in DNA replication, repair, and recombination, which ultimately leads to bacterial cell death.^58^ Since CIP and CN are broad-spectrum antibiotics that are active against both Gram-positive and Gram-negative bacteria and present distinct mechanisms of action, these antimicrobials were selected to compare their effects on *S. aureus* and *P. aeruginosa*, both alone and in combination with CNTs.

**Figure 5:**
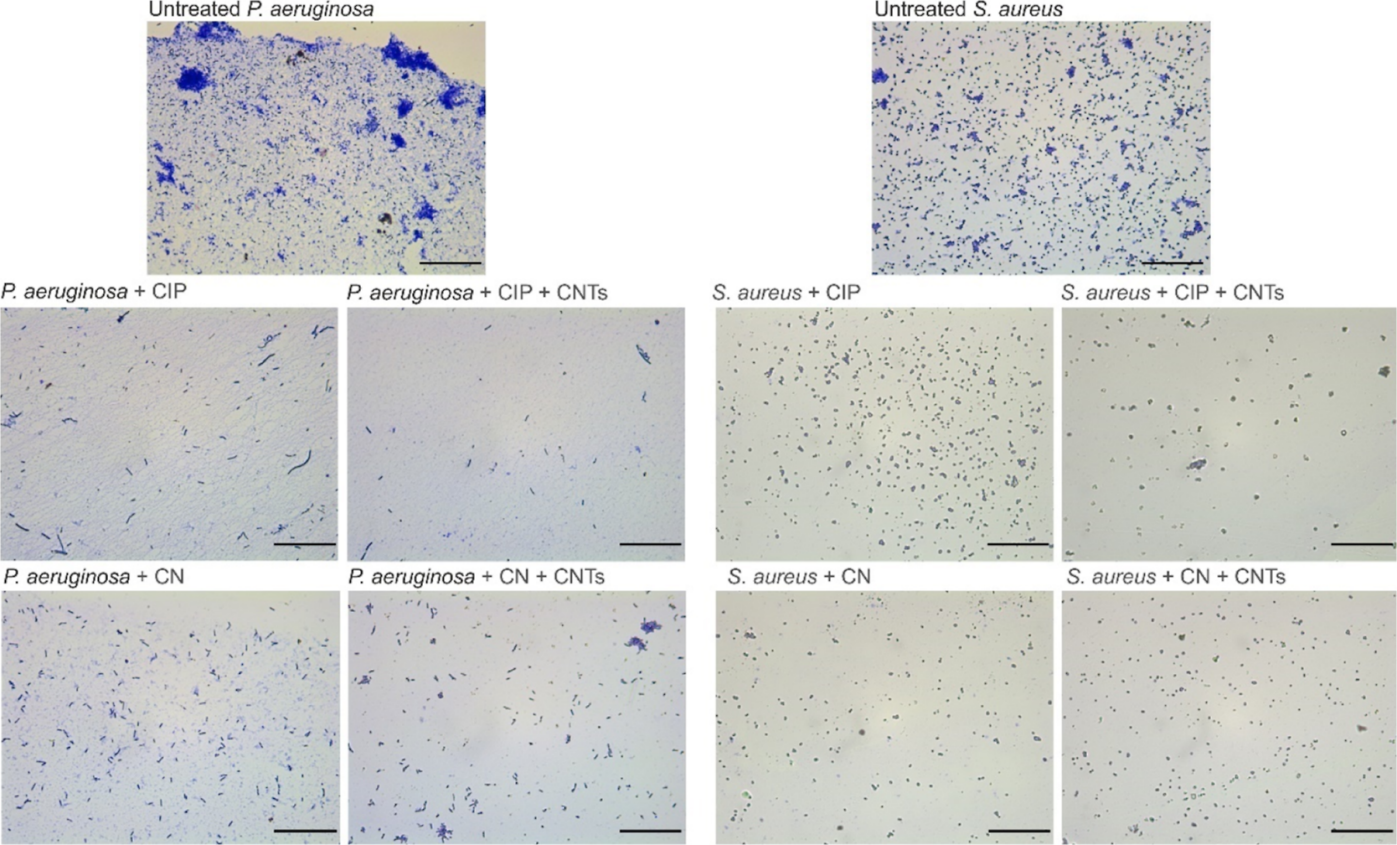
Brightfield microscopy images of *P. aeruginosa* and *S. aureus* planktonic cells treated with CNTs, alone or in combination with CIP or CN. Untreated samples were used as a control. Scale bars: 40 μm.

In Figure 5, we provide examples of the acquired brightfield images of *P. aeruginosa* and *S. aureus* cells untreated and treated with CNTs, alone or in combination with CIP or CN. For *P. aeruginosa*, the number of bacterial microcolonies is greatly reduced upon treatment with CIP and CN, and, to an even further extent, upon the addition of CNTs. For *S. aureus*, a similar trend could be observed for CIP, but no significant differences could be observed between CN alone and the combination CN + CNTs. For both *P. aeruginosa* and *S. aureus*, CN showed a lesser effect than CIP.

**Figure 6:**
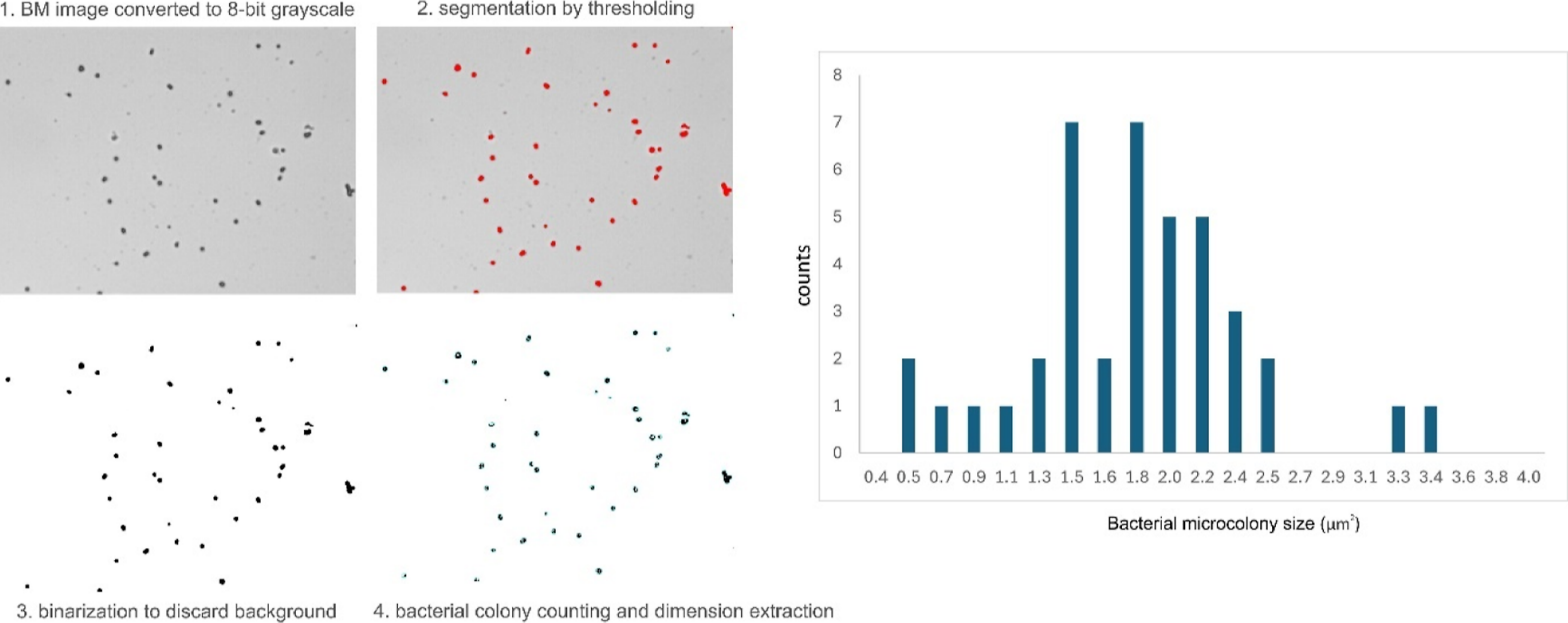
Workflow for quantitative evaluation of brightfield microscopy (BM) images collected on *P. aeruginosa* and *S. aureus* samples in the evaluated configurations.

To mine quantitative data from the collected microscopy pictures, the size of the bacterial aggregates on the acquired images was evaluated using a semiautomated image processing method implemented in Fiji (version 1.54g), as illustrated in Figure 6. Briefly, three brightfield microscopy images collected at 3648 × 2736 pixels were selected for each evaluated configuration. These were split into 4 equal tiles of 1824 × 1368 pixels, to alleviate inhomogeneity bias, resulting in 12 images per considered configuration. These were converted from 24 bit RGB format into 8 bit grayscale format. Next, on the grayscale version, we applied an intensity threshold mask to segment the bacteria and discard the background. The segmented image was then binarized, rendering the bacteria black and the background white. Using Fiji’s “Analyze Particle” function, individual bacterial structures were identified, and subsequently, quantitative data on the size of bacterial aggregates were obtained. The results were then used to calculate the histogram of bacterial aggregates dimensions.

**Figure 7:**
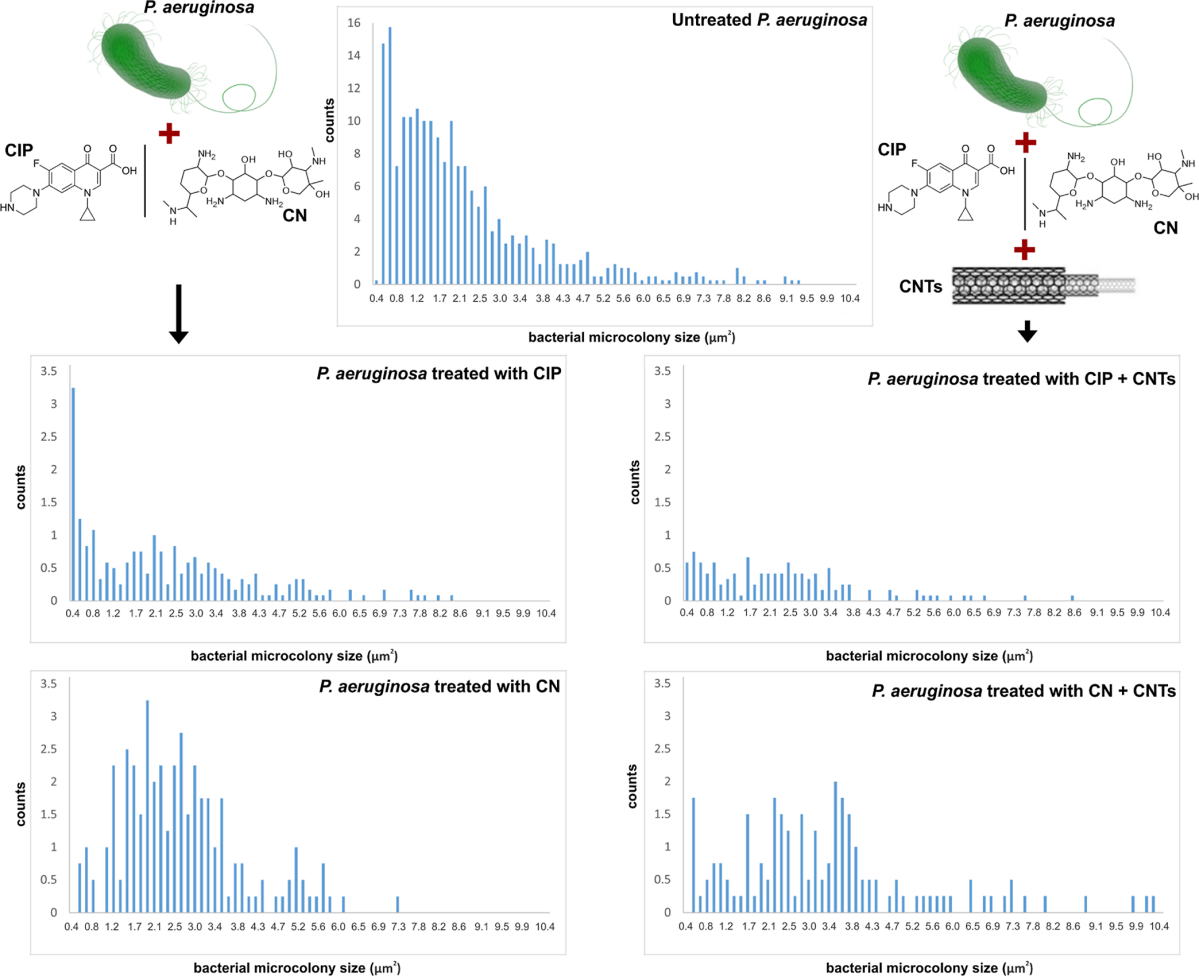
Quantitative analysis of the effects against *P. aeruginosa* planktonic aggregates of CIP, CIP + CNTs, CN, and CN + CNTs, in terms of histograms of microcolony size (calculated from brightfield microscopy images). Drawing of *P. aeruginosa*, credit to DataBase Center for Life Science (DBCLS)/ Wikimedia Commons/CC-BY-4.0.

**Figure 8:**
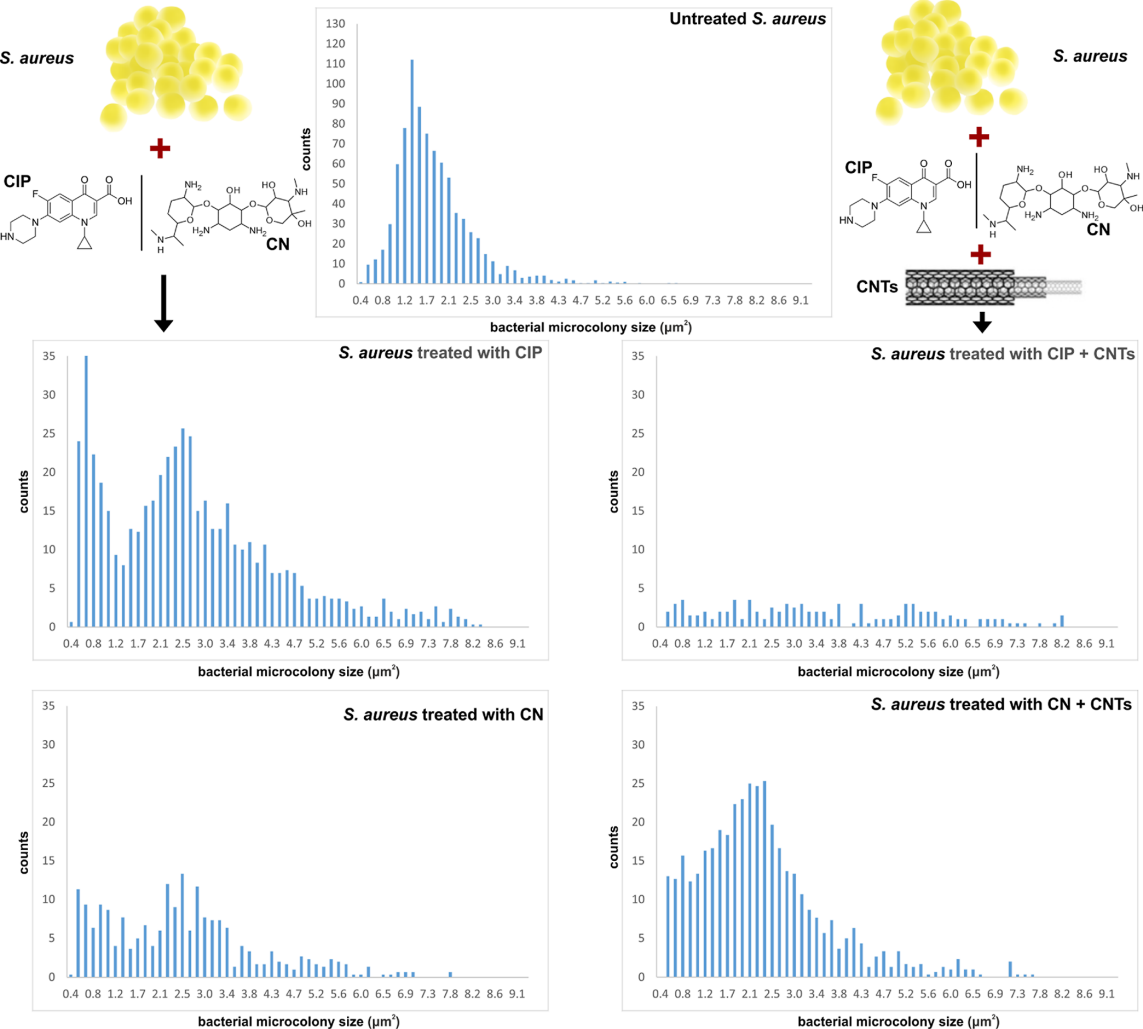
Quantitative analysis of the effects against *S. aureus* planktonic aggregates of CIP, CIP + CNTs, CN, and CN + CNTs, in terms of histograms of microcolony size (calculated from brightfield microscopy images). Drawing of *S. aureus*, credit to DataBase Center for Life Science (DBCLS)/ Wikimedia Commons/CC-BY-4.0.

In Figures 7 and 8, we present the results of this pipeline applied to the quantification of *P. aeruginosa* and *S. aureus* aggregates cells for the various treatment configurations. The results are presented in terms of histograms of microcolony size, extracted from the brightfield images. A higher ratio between the number of histogram bins corresponding to low microcolony sizes and high microcolony sizes indicates a greater dispersal effect of the evaluated configuration. For *P. aeruginosa*, treatment with CIP has a significant effect against cell aggregation, augmented by the addition of CNTs (Figure 7). Although the effect of CN and CN + CNTs is less compared to CIP and CIP + CNTs, the same trend can be observed.

Concerning *S. aureus*, treatment with CIP also shows a significant effect on the cell microcolony dimension, which is further enhanced by the addition of CNTs (Figure 8). The results for CN are less intuitive. CN demonstrates a dispersal activity slightly less pronounced than that of CIP. However, the addition of CNTs leads to reduced dispersal efficiency. Treating both the bacterial species with EtOH-dispersed CNTs yielded results that ranged between those obtained for the treatment with antibiotics and antibiotics + CNTs (Figure S2).

### Nanoscale Investigation of CNTs’ Effects on Bacterial Cells

**Figure 9:**
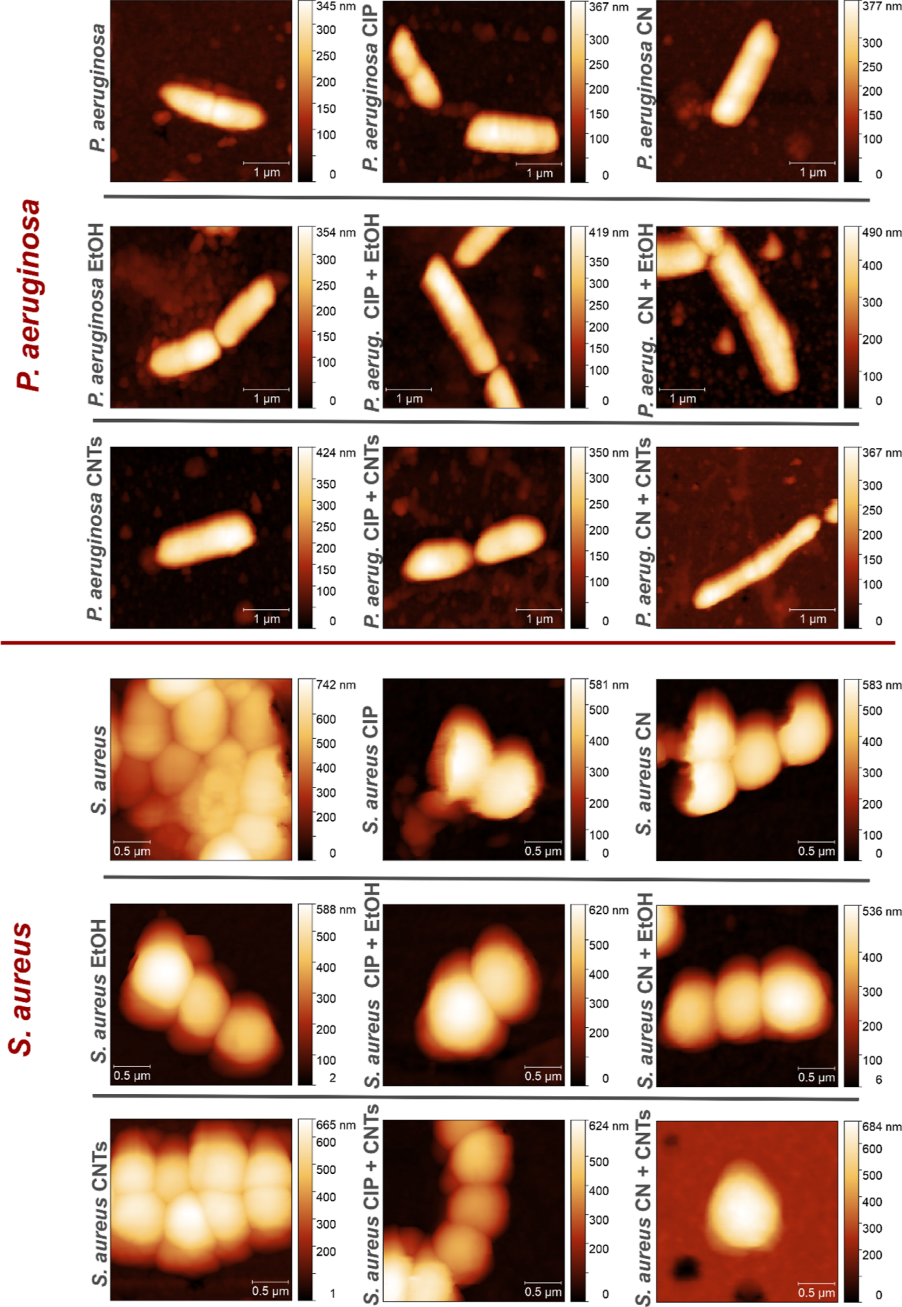
AFM images collected on *P. aeruginosa* and *S. aureus* bacterial cells interacting with CIP, CN, EtOH, and CNTs, in different combinations.

Examining cells directly under high magnification is among the most effective methods for evaluating toxicity effects in bacterial cells induced by nanomaterials. AFM is generally acknowledged as a powerful technique for studying bacteria at the nanoscale, providing information on their structure, mechanics, and interactions.^59−62^ Here, we employed AFM to identify potential morphological changes of the bacterial cells corresponding to the two tested species, following their interactions with EtOH, antibiotics, and CNTs (at 0.5 × MIC). In Figure 9, we present representative AFM images of the *P. aeruginosa* and *S. aureus* cells treated with various combinations of CN, CIP, CNTs, and EtOH. As support for interpreting the AFM images, we should remind that *P. aeruginosa* is a rod-shaped bacterium, with cells typically straight or slightly curved rods. The AFM images collected in our experiment for *P. aeruginosa* bacterium interacting with CIP, CN, EtOH, and CNTs align with this general knowledge of the shape and size of *P. aeruginosa* cells. Although the cells are not identical, we could not observe any significant morphological differences between the control group and the groups treated with CIP, CN, EtOH, and CNTs. On the other hand, *S. aureus* presents spherical cells that typically appear in grape-like clusters. The AFM images collected in our experiment for all evaluated configurations are in line with this description of the typical morphology of *S. aureus* cells. Although there are slight variations in the spherical shape and size of *S. aureus* cells (Figure 9), we did not observe significant differences that could be attributed to the interaction of *S. aureus* with CIP, CN, EtOH, or CNTs.

**Figure 10:**
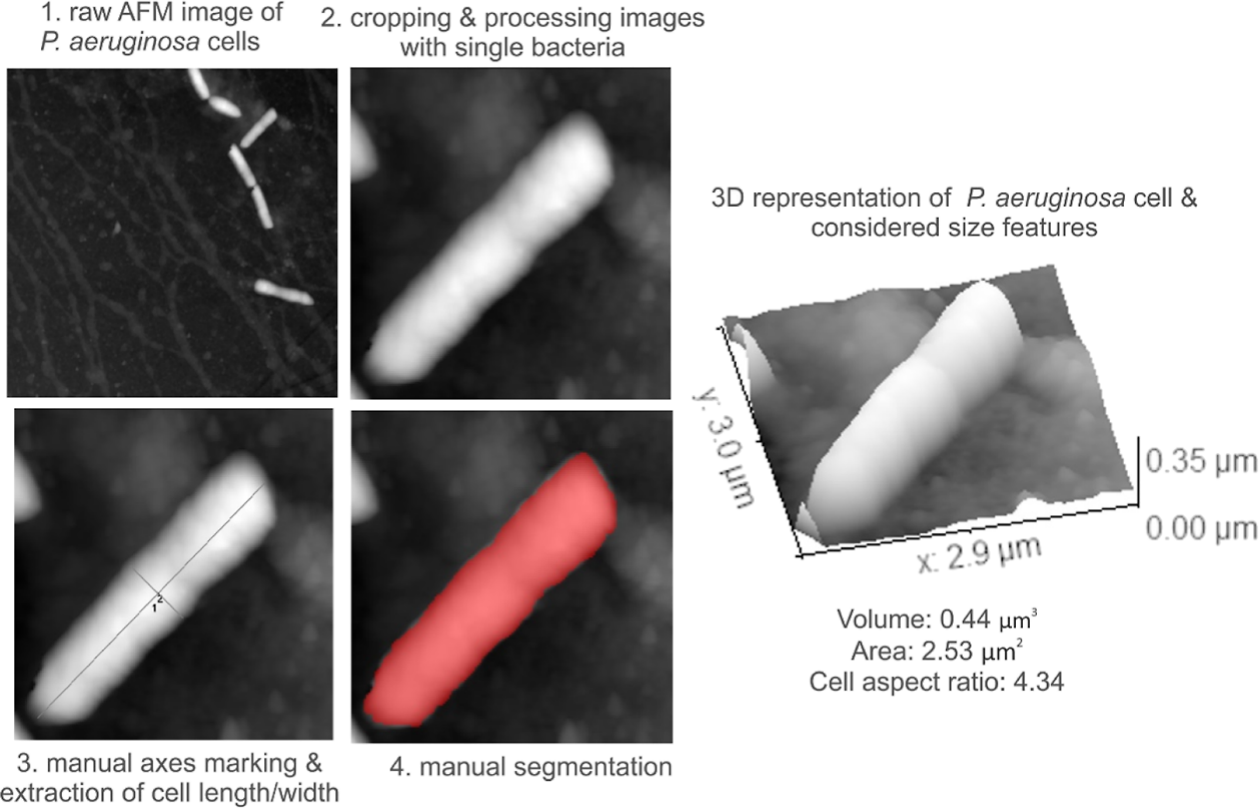
Workflow for the extraction of quantitative data from AFM images.

Although the AFM images collected for *P. aeruginosa* and *S. aureus* bacteria interacting with CIP, CN, EtOH, and CNTs did not reveal significant differences in morphology between the control group and the groups evaluated for antibacterial effects, we have further explored the morphological effects of the antibacterial configurations by segmenting the bacteria in the AFM images and calculating their volume and cell aspect ratio (major/minor axis ratio, a.k.a. cell length/width). In brief, this analysis was performed using the open-source Gwyddion software (version 2.61), a well-established tool for the processing and analysis of scanning probe microscopy data. In brief, after an AFM image was loaded in Gwyddion, it was cropped to isolate the bacteria at the individual level. Common AFM image artifacts, such as tilt effects, were corrected by mean plane subtraction, and the zero point of the image was set as the level of the substrate on top of which the bacteria were placed. We then drew perpendicular lines across the bacterium body to measure its length and width. To determine the bacterium’s surface area and volume, we manually defined a mask to segment the bacterium from the background and then we used the “Statistical Quantities” function of Gwyddion to extract the surface area and volume. For each of the considered configurations, at least 10 bacterial cells were considered. This workflow is summarized in Figure 10.

The quantitative parameters extracted were used for statistical analysis. To this end, we used one-way ANOVA followed by the Dunnett multiple comparisons test performed in Prism 10 (GraphPad Software, USA). The normality of the samples was assessed using the Shapiro–Wilk test.

The axis ratio is a widely used measure to describe the shape of bacterial cells. Different bacterial species and strains can have various shapes and sizes, and changes in the axis ratio may occur under different growth conditions or stressors. For example, bacterial cells may elongate or change shape in response to environmental factors. Under stress conditions, bacteria may exhibit alterations also in terms of their volume. For example, some bacteria may undergo cell shrinkage or elongation in response to environmental stressors such as nutrient limitation, changes in temperature, or exposure to toxins. In contrast, certain stress conditions might lead to an increase in cell volume as a part of the bacterial stress response.^63^

**Figure 11:**
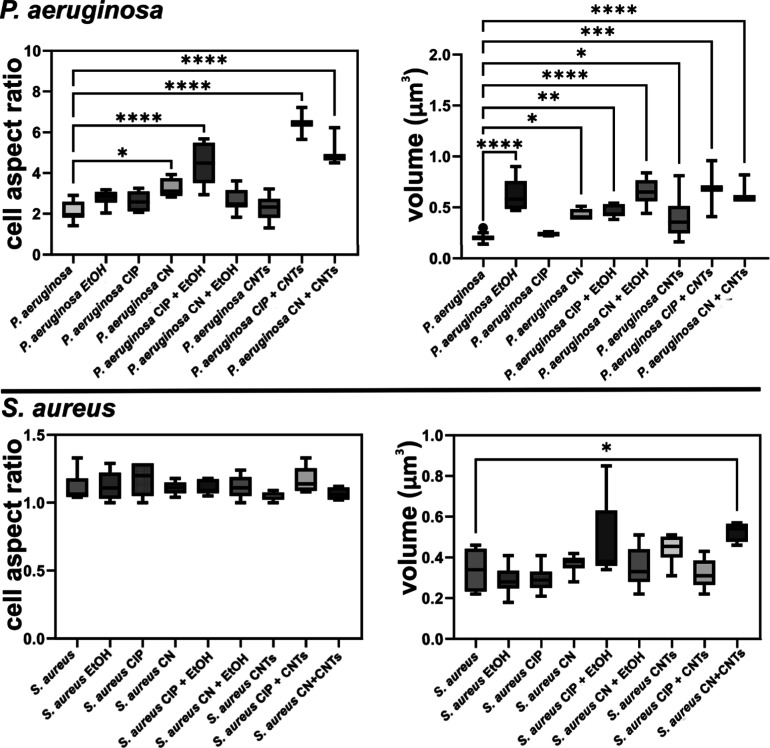
Morphological analysis on the axis ratio and volume of bacterial cells segmented from AFM images.

Figure 11 displays the results obtained using the workflow for data extraction from AFM images. In the case of *P. aeruginosa*, the axis ratio showed an increase for the test samples compared to the control sample. Statistically significant increases were obtained for *P. aeruginosa* treated with CN, EtOH + CIP, CNT + CN, and CNT + CIP, with the highest increase in the cell aspect ratio obtained for CNT + CIP. Except for the *P. aeruginosa* cells treated with CIP, all the samples demonstrate a statistically significant increase in the bacterial volume, with the highest increment of more than 3 times observed for *P. aeruginosa* cells treated with EtOH, EtOH + CN, CNT + CIP, and CNT + CN. For the *P. aeruginosa* test samples, the highest increase in both ratio and volume was obtained using the combinations CNT + CIP and CNT + CN.

In the case of *S. aureus*, no statistically significant difference between treated and untreated samples was obtained regarding the axis ratio. While the bacteria cells seem to remain unchanged for the test samples, the only statistically significant increase was observed for the volume of *S. aureus* cells treated with the CNT + CN combination.

## Conclusions

In this study, we investigated the antibacterial properties of CNTs with diameters ranging from 50 to 150 nm against two opportunistic pathogens, which are among the most antibiotic-resistant nosocomial pathogens. These include a Gram-positive species, *S. aureus*, and a Gram-negative one, *P. aeruginosa*. The initial disk diffusion assay did not reveal any antibacterial effects of the tested CNTs. However, recognizing the limitations of diffusion assays on the assessment of the antibacterial activity of high-molecular-weight compounds (such as CNTs), we employed alternative evaluation methods. By using the microdilution assays, the tested CNTs were found to be ineffective against *P. aeruginosa*, while exhibiting a slight antistaphylococcal activity, with a MIC of 0.125 mg/mL. These results were confirmed by comparing the CNTs’ activity on *S. aureus* growth and culturability at different inhibitory and subinhibitory concentrations.

Further investigations focused on the synergistic interaction between CNTs and a panel of antibiotics routinely used for the antibiogram determination. In *P. aeruginosa*, antibiotics TZP, FEP, CN, and DOR exhibited a synergistic effect with CNTs. For *S. aureus*, a synergistic interaction was observed with the antibiotic FOX. Brightfield microscopy was employed to visualize the effects of CNTs, either alone or in combination with the two broad-spectrum antibiotics CIP and CN, on the *P. aeruginosa* and *S. aureus* planktonic aggregates. Qualitative analysis, along with a novel implemented approach based on the histograms of microcolony size calculated on the collected microscopy images, demonstrated the dispersal effects of CIP, CIP + CNT, CN, and CN + CNT for both bacterial species.

Additionally, AFM was employed to explore potential morphological changes in bacterial cells of the two species after exposure to EtOH, antibiotics exhibiting synergistic activity with CNTs, and CNTs alone. While significant morphological differences were not initially discernible through a qualitative examination of the obtained images, a dedicated pipeline for AFM image analysis unveiled variations in cellular axis ratio and volume, especially in *P. aeruginosa* cells. These findings suggest morphological alterations induced by the antimicrobial compounds.

In conclusion, although the configuration of generated CNTs requires high concentrations or combination with other antimicrobial agents, thereby precluding direct use in clinical practice, the combination with EtOH and high-concentration antibiotics could be utilized as a disinfectant agent, paving the way for new potential applications of these carbon-based materials.

## Supporting Information Available

Kirby–Bauer disk-diffusion assay of CNTs; representative brightfield microscopy images and quantitative analysis of the effects against *P. aeruginosa* and *S. aureus* planktonic aggregates of EtOH-dispersed CNTs; and diameters of ZOI for *P. aeruginosa* and *S. aureus* of antibiotics alone and in combination with CNTs or EtOH (PDF)
