# Supplementary material for: Antibacterial Interactions of Ethanol-Dispersed Multiwalled Carbon Nanotubes with Staphylococcus aureus and Pseudomonas aeruginosa
Source: ACS Omega. 2024 Jul 9;9(31):33751–64. doi: 10.1021/acsomega.4c03044 (PMC11307305; doi:10.1021/acsomega.4c03044)
Supplement: Supplementary file 1 — ao4c03044_si_001.pdf [file ao4c03044_si_001.pdf]

Antibacterial Interactions of Ethanol-Dispersed Multi-Walled Carbon Nanotubes with *Staphylococcus aureus* and *Pseudomonas aeruginosa*

Supplementary Information

Mihaela Asaftei[1,2+], Massimiliano Lucidi[3,4,+], Stefan Razvan Anton[1], Aikaterini-Flora Trompeta[5], Radu Hristu[1], Denis E. Tranca[1], Efstathios Fiorentis[1], Cristina Cirtoaje[1], Veronica Lazar[2], George A. Stanciu[1], Gabriella Cincotti[6], Paola Ayala[7], Costas A. Charitidis[5,*,] Alina Holban[2],*, Paolo Visca[3,]*, Stefan G. Stanciu[1,]*

[1]Center for Microscopy-Microanalysis and Information Processing, National University of Science and Technology Politehnica Bucharest, 313 Splaiul Independentei, 060042, Bucharest, Romania

[2]Department of Microbiology and Immunology, Faculty of Biology, Research Institute of the University of Bucharest, University of Bucharest, 060101 Bucharest, Romania

[3]Department of Science, Roma Tre University, Viale G. Marconi 446, 00146 Rome, Italy

[4]NBFC, National Biodiversity Future Center, piazza Marina 61, 90133 Palermo, Italy.

[5]Research Lab of Advanced, Composite, Nano-Materials and Nanotechnology (R-NanoLab), School of Chemical Engineering, National Technical University of Athens, 9 Heroon Polytechniou, 15773 Athens, Greece

[6]Department of Engineering, Roma Tre University, Viale G. Marconi 446, 00146 Rome, Italy;

[7]Faculty of Physics, University of Vienna, Boltzmanngasse 5, A-1090, Vienna, Austria

[+]These authors contributed equally to this work

*Corresponding authors:

Costas A. Charitidis: charitidis@chemeng.ntua.gr
Alina Maria Holban: alina.m.holban@bio.unibuc.ro
Paolo Visca: paolo.visca@uniroma3.it
Stefan G. Stanciu: stefan.g.stanciu@upb.ro

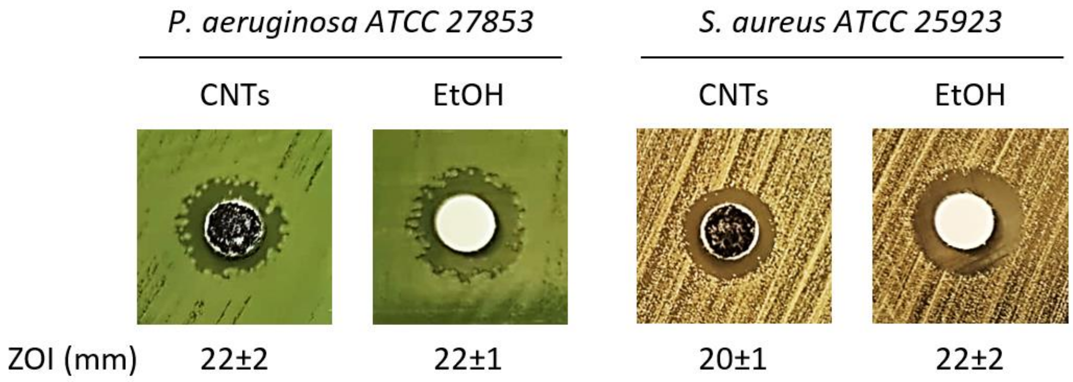

Fig. S1. Kirby-Bauer disk-diffusion assay of CNTs. Blank disks were soaked with 20 µl of CNTs (2 mg/mL in EtOH) or 20 µl of EtOH. The corresponding ZOI diameter is reported for each strain. The indicated ZOI diameters are the results of three independent experiments ± SD.

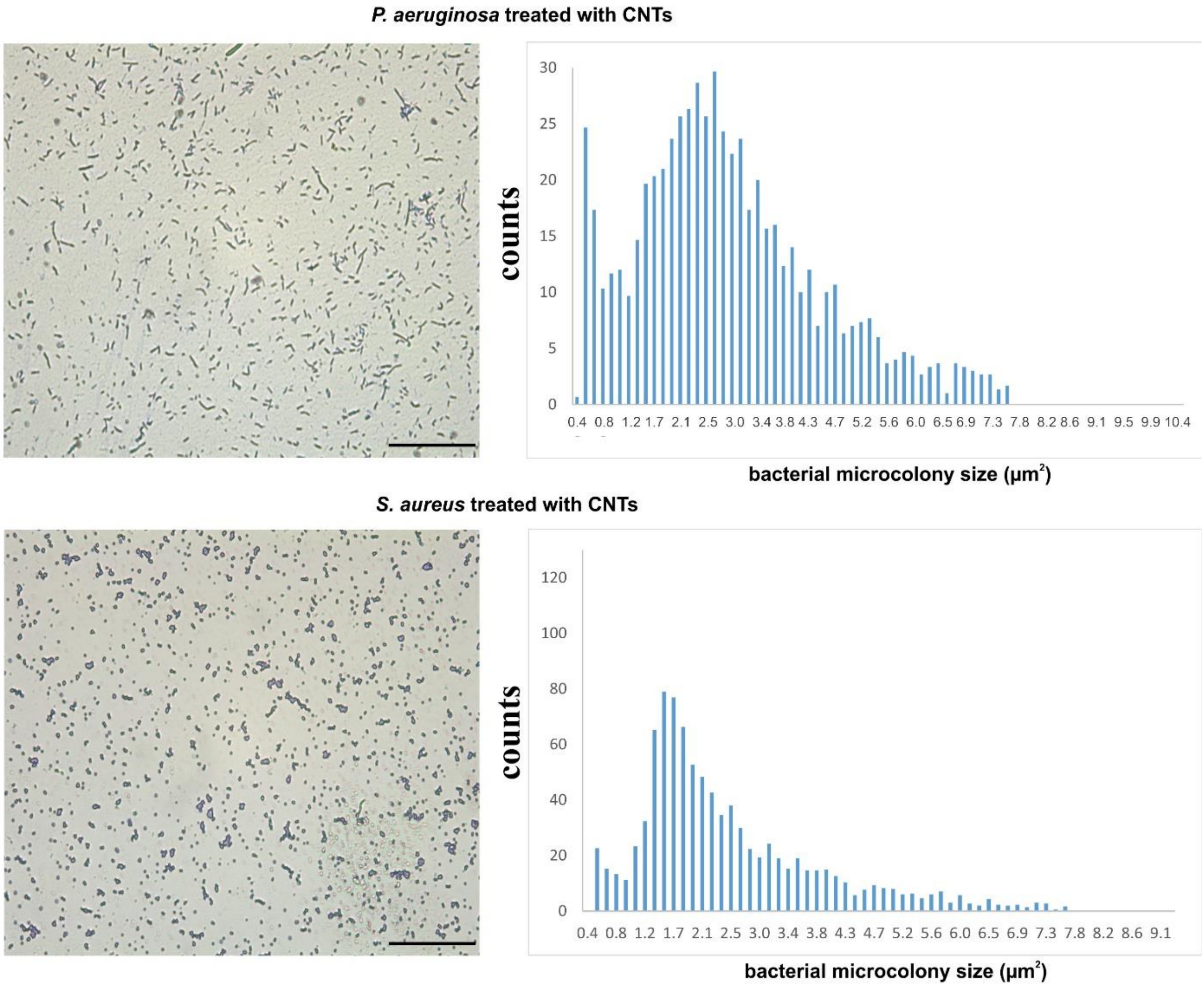

Fig. S2 Representative brightfield microscopy images and qquantitative analysis of the effects against *P. aeruginosa (top) and S. aureus (bottom)* planktonic aggregates of EtOH dispersed CNTs. The histograms of bacterial microcolony size on the right are calculated from twelve brightfield microscopy images.

Table S1. Diameters of ZOI for *P. aeruginosa* of antibiotics alone and in combination with CNTs or EtOH.

	IMP (10 µg)	TZP (110 µg)	CAZ (30 µg)	FEP (30 µg)	ATM (30 µg)	MEM (10 µg)	CN (120 µg)	CIP (5 µg)	TOB (10 µg)	AK (30 µg)	DOR (10 µg)
**Antibiotic + CNTs**	28	**36**	28	**32**	32	36	**36**	38	26	26	**44**
**Antibiotic + EtOH**	28	35	29	31	34	36	32	38	26	26	40
**Antibiotic**	28	30	24	30	36	34	30	40	25	26	40

Values in bold indicate synergistic activity. Abbreviations: imipenem (IMP); piperacillin/tazobactam (TZP); ceftazidime (CAZ); cefepim (FEP); aztreonam (ATM); meropenem (MEM); gentamicin (CN); ciprofloxacin (CIP); tobramycin (TOB); amikacin (AK); doripenem (DOR). The antibiotic dosage for each disk is reported in brackets. Data are the result of a single assay.

Table S2. Diameters of ZOI for *S. aureus* of antibiotics alone and in combination with CNTs or EtOH.

	FOX (30 µg)	AZM (15 µg)	CN (10 µg)	CIP (5 µg)	CPT (30 µg)	P (10 µg)	TE (30 µg)	DA (2 µg)	E (15 µg)	RD (5 µg)	SXT (25 µg)	LZD (10 µg)
**Antibiotic + CNTs**	**18**	9	25	32	36	11	17	32	13	37	32	30
**Antibiotic + EtOH**	16	9	26	32	36	12	18	32	16	37	32	30
**Antibiotic**	16	6	22	30	34	12	16	30	6	36	32	30

Values in bold indicate synergistic activity. Abbreviations: cefoxitin (FOX); azithromycin (AZM); gentamicin (CN); ciprofloxacin (CIP); ceftarolin (CPT); penicillin G (P); tetracycline (TE); clindamycin (DA); erythromycin (E); rifampicin (RD); trimethoprim/sulfamethoxazole (SXT); levofloxacin (LZD). The antibiotic dosage for each disk is reported in brackets. Data are the result of a single assay.
